# Improving tumble turn performance in swimming—the impact of wall contact time and tuck index

**DOI:** 10.3389/fspor.2022.936695

**Published:** 2022-07-22

**Authors:** Sina David, Tamara Grove, Myrna v. Duijven, Paul Koster, Peter J. Beek

**Affiliations:** ^1^Department of Human Movement Sciences, Vrije Universiteit Amsterdam, Amsterdam Movement Sciences, Amsterdam, Netherlands; ^2^InnoSportLab De Tongelreep, Eindhoven, Netherlands

**Keywords:** free style, front crawl, optimization, prediction, flip turn

## Abstract

Race time can be shortened by improving turn performance in competitive swimming, but this requires insight into the optimal turn technique. The aim of the present study was to examine the effect of Wall Contact Time (WCT) and Tuck Index on tumble turn performance and their interrelations by experimentally manipulating both variables, which has not been done in previous research. Eighteen Dutch national level swimmers (FINA points 552 ± 122) performed tumble turns with three different WCTs (shorter, preferred, longer) and three different Tuck Indices (higher, preferred, lower), which were recorded by four underwater cameras and a wall-mounted force plate. Linear kinematic and kinetic variables, including the approach velocity (V_in_), wall adaptation time (T_adapt_), percentage of active WCT (aWCT), peak push-off force (F_Peak_) and exit velocity (V_exit_), were extracted from the recordings and analyzed statistically, using the 5 m round trip time (5mRTT) as performance measure. The results indicated that the WCT should be sufficiently long to generate a high push-off force at the end of wall contact when the body is in a streamlined position. This led to a significantly shorter 5mRTT than a shorter or longer WCT. A linear mixed effect model yielded negative significant effects of WCT (−4.22, *p* < 0.001), F_Peak_ (−2.18, *p* = 0.04), V_in_ (−4.83, *p* = 0.02), T_adapt_ (−2.68, *p* = 0.002), and V_exit_ (−9.52, *p* < 0.001) on the 5mRTT. The best overall turning performance was achieved with a Tuck Index of 0.7, which suggests that some of the participating swimmers could benefit from adapting their distance to the wall while turning, as was exemplified by calculating the optimal Tuck Index for individual swimmers. These results underscore the importance of WCT and Tuck Index vis-à-vis tumble turn performance, as well as their interrelations with other performance determining variables in this regard.

## Introduction

The margin between winning a gold or silver medal on the 100 m freestyle during the Tokyo Olympic Games 2021 was 0.06 s. With such small differences, every opportunity for improvement should be exploited to optimize the chances of winning (Arellano et al., 1994; Morais et al., 2019). In general, performance improvement can be achieved in any of the four components of a swimming race, i.e. starting, free swimming, turning and finishing. Born et al. (2021) reported that the total turn time, defined from 5 to 10 m out of the wall, increases with the race distance, as does its contribution to the total swim time (Morais et al., 2019). Accumulated over entire race events, the time spent turning represents about 20 % of the total race time in the 100 m freestyle for elite male sprinters on the long course (i.e., 50 m pool) (Morais et al., 2019), and more than 40 % on the short course (i.e., 25 m pool). Arellano et al. reported a positive relationship between race time and total turn time with correlation coefficients ranging between 0.8 and 0.9 for various race distances (Arellano et al., 1994). One reason for such a high contribution to swimming performance is the high velocity that is achieved after a forceful push-off from the wall, which is significantly higher than the average swimming velocity (Shimadzu et al., 2008; Veiga and Roig, 2017). This implies that improving turning performance will not only result in a reduction of the overall race time but also in a better preparation for the next lane lap. Turning can therefore be considered a major determinant of swimming performance (Born et al., 2021) with substantial potential for improving the overall race result.

The overall turn performance is usually expressed as the cumulative duration of the approach, rotation, wall contact, glide, underwater propulsion, and stroke resumption (Puel et al., 2010). However, to identify those parts of the turn that are (most) amenable for improvement, it is necessary to split up the action sequence and examine the effects of specific performance determining variables. A major division can be made between the approach to and exit from the wall (Veiga et al., 2014). The approach to the wall depends on the swimmer's velocity (V_in_) and the time needed to rotate around the transverse axis and place their feet on the wall, the so-called adaptation time (T_adapt_). An effective push-off force, an appropriate amount of time spent on the wall and a properly streamlined position during the push-off and the subsequent glide phase are essential to a wall action resulting in a high turn performance (Lyttle et al., 1998, 1999; Mason and Cossor, 2001).

The aforementioned performance-determining variables of the tumble turn have been amply studied in previous research. The peak push-off force (F_Peak_) has been considered the variable with the highest influence on the tumble turn time (Blanksby et al., 1996; Araujo et al., 2010). However, the generation of a high F_Peak_ was reported to be closely linked to both the Wall Contact Time (WCT) and Tuck Index (Blanksby et al., 1996; Araujo et al., 2010; Cossor et al., 2014; Skyriene et al., 2017). The WCT is defined as the time between the first wall contact of the swimmers' feet and the end of wall contact. Several studies have shown that a shorter WCT is related to a higher F_Peak_, resulting in faster turn times (Blanksby et al., 2004; Pereira et al., 2006; Araujo et al., 2010). However, a longer WCT allows the swimmer time to produce the F_Peak_ more toward the end of the push-off. This might be an advantage as the swimmer will be in a more streamlined position during this phase of the action such that the produced force will result in higher acceleration and exit velocity (V_exit_) due to a lower peak drag force (Lyttle et al., 1999). Since the WCT is actively used to generate this push-off force, the amount of active WCT (aWCT) should be considered when increasing the overall WCT.

The WCT correlates negatively with the Tuck Index (Blanksby et al., 2004; Pereira et al., 2006), implying that a higher Tuck Index is associated with a shorter WCT (Blanksby et al., 2004; Pereira et al., 2006). The Tuck Index is defined as the minimal distance of the hip from the wall expressed as a percentage of the trochanter major height. A Tuck Index of for instance 0.60 implies that the closest distance from the trochanter major to the wall corresponds to 60 % of the swimmer's leg length. Having the lower limbs in a less bent position implies that it takes less time to extend them. This can save time and may thus result in faster swim times. A Tuck Index of 0.57 ± 0.17 was reported in age-group swimmers (i.e., of 11.8 ± 0.7 years old) (Blanksby et al., 1996). This finding was replicated in subsequent studies, in which Tuck Indices ranging from 0.56 to 0.71 were reported (Cossor et al., 1999; Blanksby et al., 2004; Patz, 2005; Prins and Patz, 2006; Skyriene et al., 2017; Smithdorf, 2018). However, the literature also contains some inconsistent findings. Two studies reported a negative relationship between Tuck Index and turn performance (Cossor et al., 1999; Blanksby et al., 2004), while others reported a positive relationship (Cossor et al., 2014; Skyriene et al., 2017). This contradiction can be due to the large range of knee joint angles (29–161°) that were reported (Araujo et al., 2010) during the tumble turn. Besides reducing the time to extend the lower limbs, a higher Tuck Index means that the swimmers turn further away from the wall and thus have to cover a shorter distance swimming. However, there is an upper limit of the Tuck Index because swimmers also have to be able to generate an effective push-off force to exit from the wall at high speed. This suggests that an optimal Tuck Index exists, which needs to be identified to improve turn performance (Nicol et al., 2019).

Although the tumble turn and its performance determining variables have been extensively investigated in previous studies, none of these studies involved experimental manipulations of those variables to assess their effects on tumble turn performance and interrelations with other performance-determining variables. Experimental manipulation of performance determining variables is required to gain further insight into the complexity of the tumble turn for at least two reasons. First of all, it provides a means to induce sufficient variation in relevant variables (both the explicitly manipulated and other relevant variables) to derive reliable and meaningful prediction models (Nicol et al., 2019), an option that is precluded when focusing solely on the preferred turn technique. In addition, it allows to examine the performance response of each individual swimmer to the manipulation, which can then be interpreted in relation to more general results, such as a prediction model, to identify aspects of the tumble turn that might be improved through technique refinements in dedicated training sessions.

Against this background, the present study aimed to examine the effect of the WCT and Tuck Index on tumble turn performance, defined as the 5 m round trip time (5mRTT). This definition was chosen because it limits the influence of free swimming while still allowing to determine the speed underwater (Blanksby et al., 1996; Pereira et al., 2015). For this reason, the 5mRTT is the most commonly used performance measure in pertinent literature and the one recommended by Silveira et al. (2011) to describe the turning performance in sub-elite swimmers. A longer WCT was hypothesized to result in a shorter 5mRTT due to the improved position of the body during the push-off from the wall. Furthermore, a longer aWCT was hypothesized to be beneficial in generating a high F_Peak_ at an appropriate time, resulting in a higher V_exit_. By the same logic, a shorter WCT was hypothesized to result in a higher F_Peak_, as found in previous studies, but not in a shorter 5mRTT. Based on previous results, it was further hypothesized that, within the reported Tuck Index range of 0.55 to 0.70, a negative relationship exists between the Tuck Index and WCT on the one hand and a positive relationship between the Tuck Index and F_Peak_ on the other hand. Finally, it was hypothesized that the relationship between the Tuck Index and turn performance can be explained by a quadratic estimation function, based on the assumption that an optimal Tuck Index exists for each swimmer. In the context of examining this last hypothesis, an attempt was made to identify the optimal Tuck Index for the participating swimmers.

## Materials and methods

Eighteen Dutch national-level swimmers (eight male, ten female, see Table 1 for further details) participated in the experiment. The participants or their legal guardians in case they were 16 years of age or younger signed an informed consent form before participation. To assess the performance level of the swimmers, who were all freestyle competitors, their personal best times and corresponding FINA points for the 100 m freestyle were collected. The FINA points were calculated based on the swimmers' personal best times as of July 2021 and expressed relative to the world record for male and female swimmers separately, up to a maximum of 1,000 FINA points. Personal best and FINA points are reported in Table 1.

**Table 1:** Mean ± standard deviation of the participant's age, mass, height, leg length, and performance level.

	**Age (years)**	**Mass (kg)**	**Height (m)**	**Leg length (m)**	**Personal Best (s)**	**FINA**
All (*N* = 18)	19.0 ± 4.4	68.6 ± 12.7	1.80 ± 0.07	0.93 ± 0.05	60.97 ± 4.6	552 ± 122
Male (*N* = 8)	18.3 ± 5.2	70.3 ± 14.8	1.85 ± 0.08	0.96 ± 0.05	60.31 ± 6.49	499 ± 153
Female (*N* = 10)	19.4 ± 4.0	67.3 ± 11.4	1.76 ± 0.04	0.91 ± 0.04	61.66 ± 2.51	595 ± 74

The experiment was conducted in the 50 m long training pool of swim center De Tongelreep at Eindhoven, also known as the Pieter van den Hoogeband swimming pool. After having arrived at the center and signed the informed consent form, the swimmers performed a standardized warm-up routine of ~10 min, including two practice turns at high speed. Subsequently, a marker was attached to the trochanter major of the right femur to record the position of the hip during the turn trials proper.

**Figure 1:**
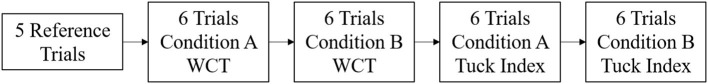
Schematic overview of the experimental protocol.

In total, four test days were held within 1 week to acquire the data for this study. All swimmers performed 29 turns in total during a single measurement session, including the turns with the manipulations of the WCT and Tuck Index, respectively. In performing those turns, the swimmers were instructed to start at about 15 m from the wall, reach 100 m race speed at about 5 m before the wall and continue swimming at 100 m race speed until 15 m out of the wall. The first 5 turns of the measurement session were executed in a preferred manner by the swimmer. These trials provided the preferred WCT and Tuck Index, respectively, which served as the reference for the manipulations that were to follow. In the next 12 trials, the swimmers were invited to perform 6 turns with a 25 % shorter (short) and 6 turns with a 25 % longer (long) WCT than in the reference trials (see Figure 1 for the experimental protocol). The order in which the short and long trials were performed was counterbalanced over the participants. Similarly, in the last 12 trials, the swimmers were invited to perform 6 turns each in which they initiated the turn at least 15 % closer (close) and at least 15 % further away (far) from the wall than in the reference trials. Analogous to the WCT manipulation, the order in which these trials were performed was counterbalanced over the participants. However, the trials with the WCT manipulation always preceded the trials with the Tuck Index manipulation (Figure 1). For the reference trials, the swimmers were instructed to perform the turns in their preferred manner. The short WCT trials were effectuated by asking them to turn with their feet leaving the wall as fast as possible, whereas for the long WCT turns they were asked to have their feet remaining on the wall as if to stick it. To execute the close turns, they were asked to turn closer to the wall in a way that their butt would almost touch the wall, whereas, for the far turns, they were asked to initiate the turn at a distance with having their legs almost extended when touching the wall.

To give direct feedback on the WCT, the force plate data was used. For the Tuck Index trials, the tumble turn initiation distance was used as a proxy, since the Tuck Index could only be assessed offline during the postprocessing procedure. Feedback was given after each trial about whether or not the experimental condition was met. Between trials, the swimmers could chose between an active or passive rest of 5 min to avoid fatigue. Trials were discarded as invalid if the swimmer did not hit the force plate, started the trial too early or too late, or did not meet the condition criteria.

**Figure 2:**
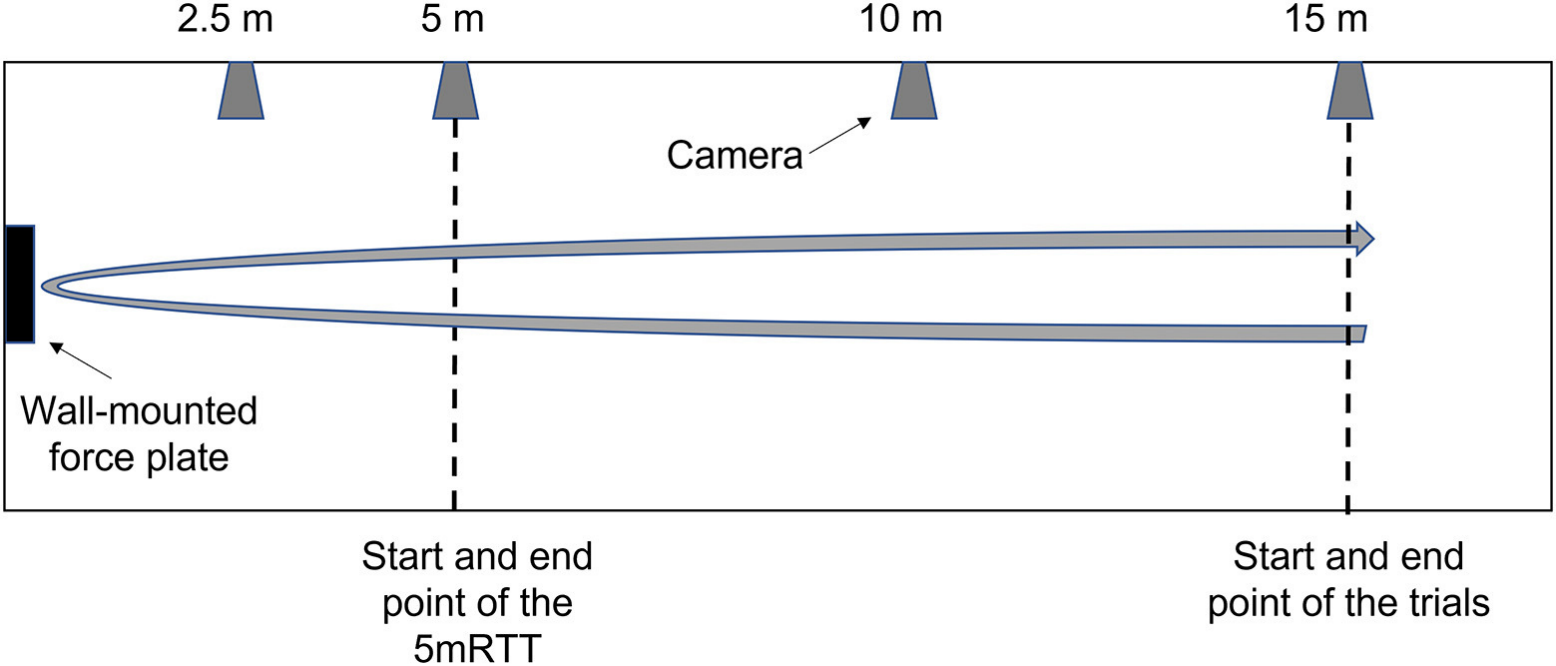
Experimental setup, displaying the location of the wall-mounted force plate (black rectangle) and the four cameras (gray trapezoids).

A 900 × 600 × 40 mm Kistler force plate (1,000 Hz, 9691A, Switzerland) embedded in the wall of the pool where push-off forces are exerted and four digital video cameras (50 Hz, scA1400-30gc, Basler, Ahrensburg, Germany) were used to record each tumble turn. The cameras and the force plate were synchronized using the software package Streampix (Norpix, Streampix 7, 2016). All video recordings were analyzed using a custom-made software program called TurnAnalyzer (Escrito sport, Eindhoven, The Netherlands). The force data was filtered using a 5 Hz low-pass third-order Butterworth filter to exclude the influence of waves and other noise. The cameras were positioned on the lateral side of the pool, at the 2.5-, 5-, 10-, and 15-m marks, respectively (see Figure 2).

**Figure 3:**
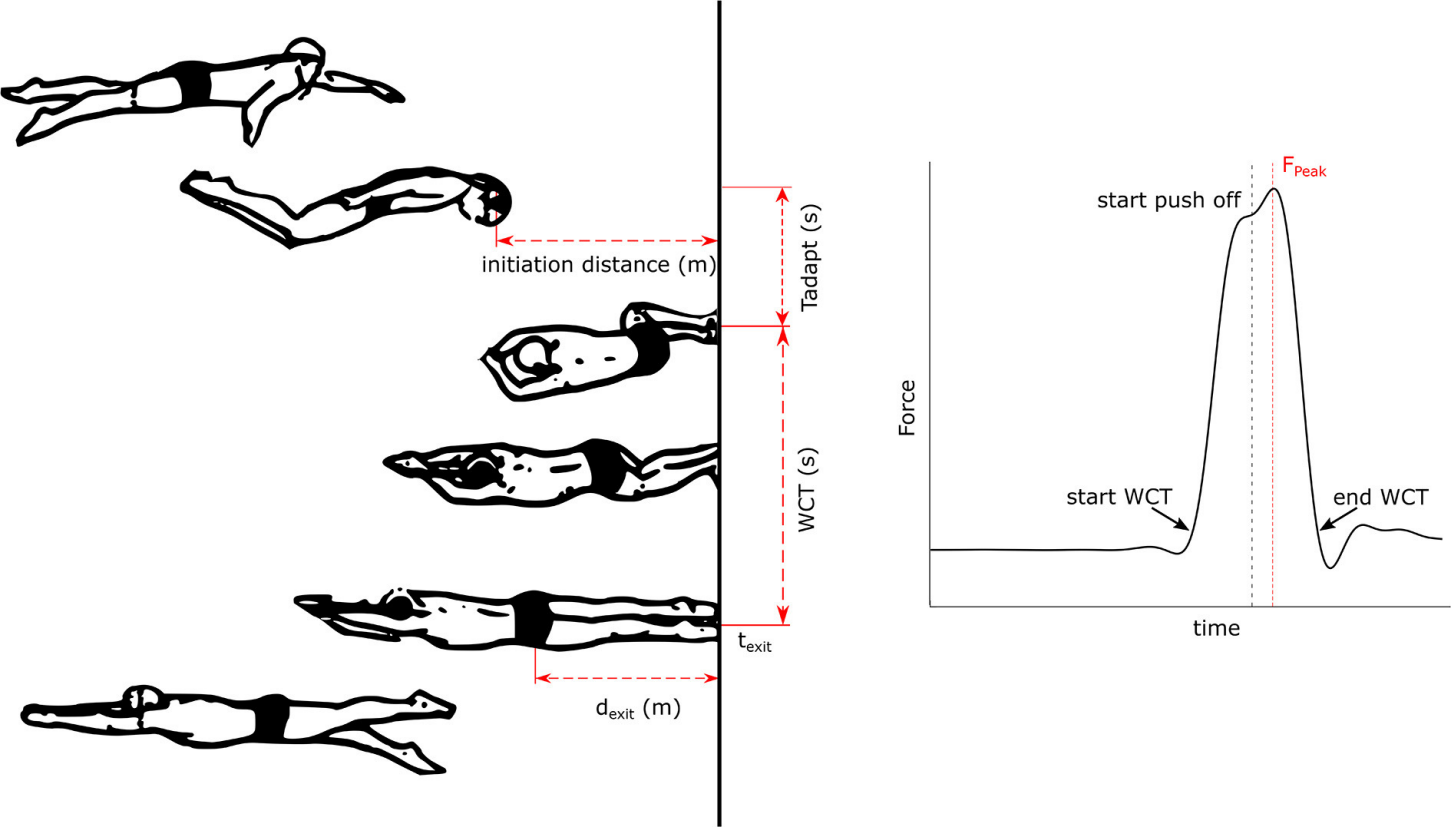
Left: Tumble turn technique [adapted from Puel et al. (2012)]: Illustrating the initiation distance, d_exit_, WCT and T_adapt_. Right: Typical force profile of a reference trial indicating the start and end of the WCT with a force > 20N, F_Peak_ and the start of the push of phase.

From each video, the Tuck Index, 5mRTT, approach and exit velocity (V_in_, V_exit_) and the adaptation time (T_adapt_), defined as the time spent by the swimmer to bring their feet to the wall, were determined (Table 2). WCT and peak Force (F_Peak_) were derived from the force plate data using a threshold of 20 N to define the first and final point of contact (see Figure 3, Table 2).

**Table 2:** Description of the variables of interest.

Tuck Index	Minimal distance of the hip from the wall expressed as a percentage of the trochanter major height
WCT (s)	Duration of the wall contact, defined by a force threshold of 20 N on the wall-mounted force plate
5mRTT (s)	Duration covering 5 m-in to 5 m-out of the wall
V_in_ (m/s)	Average approach speed between the 5 and 3 m mark before the turn.
T_adapt_ (s)	The time needed to bring the feet to the wall and measured from the time the head completely crossed the waterline until the first wall contact
F_Peak_ (*N*)	Maximum Force against the wall-mounted force plate
aWCT (%)	Active part of the WCT (see Figure 3)
5mOUT (s)	Duration from push-off from the wall until 5 m-out of the wall

The video recordings were also used to define the instant at which the WCT was partitioned into a passive and active part since this was not always visible in the force data. By combining the force plate and video data the percentage of active WCT (aWCT) was determined. The time frame of the first forward hip movement detectable on video was combined with the time frames of first wall contact and wall exit of the force plate. The instant of first wall contact until the first forward hip movement forms the passive part. Consequently, the aWCT was defined from the first forward hip movement until wall exit.

The 5mRTT was measured by the time between the swimmer's trochanter major crossing the 5 m mark on the way in and out of the wall. In addition, the 5mOUT was determined to examine the effect of the WCT and Tuck Index on the exit from the wall. The 5mOUT was defined as the time from first wall contact until the swimmer's trochanter major crossed the 5 m mark. The T_adapt_ was defined as the time required to bring the feet to the wall and starting from the time the swimmer's head completely crossed the waterline until the first wall contact (Figure 3). Various speed variables were also derived from the video recordings. The wall exit speed (V_exit_) was calculated according to:


$$\begin{array}{l} V_{exit}=\frac{\left(3-\;d_{exit}\right)}{\left(t_{3m}-\;t_{exit}\right)} \end{array}$$

where d_exit_ is the distance of the trochanter to the wall at last foot contact, t_3m_ is the instant the trochanter major passed the 3 m mark out of the wall and t_exit_ is the instant of last foot contact.

The data were analyzed using SPSS (IBM SPSS Statistics, Version, 27.0) and Matlab R2020b. To examine whether the swimmers achieved significantly different WCTs and Tuck Indices during the test conditions, 3 × 2 ANOVAs with repeated measures were performed with the experimental condition as the within-participant factor (3 levels) and sex as a between-subject factor (2 levels). Additionally, it was checked whether the manipulations resulted in differences for the 5mRTT and the F_peak_ using the same method. Bonferroni *post-hoc* tests were performed if significant main or interaction effects were found. Pearson correlation coefficients were calculated to see how the different variables were related to each other. In addition, a linear mixed effect model analysis was performed to examine the extent to which the WCT, F_Peak_, T_adapt_, V_in_, and V_exit_ accounted for the 5mRTT. To estimate the optimal Tuck Index a quadratic estimation function was used on both the entire group and the individual swimmers. To gain further insight into the effect of the manipulations of WCT and Tuck Index, the Bland-Altman values and the degree of consistency among measurements by means of pairwise intra-class correlations were calculated using the equations described in Haghayegh et al. (2020).

## Results

### Manipulation of WCT

In total, 288 out of 306 tumble turn trials were included to examine the effect of WCT on turn performance. The statistical results are reported in Table 3. The WCTs were significantly different across the experimental conditions in the expected directions, indicating that the manipulation was successful. The male swimmers showed significantly longer WCTs compared to the female swimmers. The aWCT decreased significantly from the shorter to the reference to the long WCTs, implying that the swimmers spent a longer time passively on the wall with increasing WCT. In this regard, no significant effects of sex were found. The highest F_Peak_ was generated during the short WCT trials and differed significantly from the reference trials and long contact trials.

**Table 3:** Descriptive (mean ± standard deviation) and statistical results of the 2 × 3 repeated measures ANOVA design with the manipulation condition as the within-subject factor (main effect) and sex as the between-subject factor (interaction effect).

**Manipulating WCT**	**Sex**	**Short**	**Reference**	**Long**	**F-Statistics (condition)**	**F-Statistics (Sex)**	**p and d**
WCT (s)	Male Female	0.3 ± 0.06 0.25 ± 0.06	0.37 ± 0.04 0.3 ± 0.05	0.47 ± 0.05 0.4 ± 0.09	*F*_(1.47, 23.51)_ = 96.969, *p* < 0.001, ${\text{η}}_{\text{p}}^{2}$ = 0.858	*F*_(1,16)_ = 6.287, *p* = 0.023, ${\text{η}}_{\text{p}}^{2}$ = 0.282	1 vs. 2: *p* < 0.001, *d* = 1.77, 1 vs. 3: *p* < 0.001, *d* = 2.09, 2 vs. 3: *p* < 0.001, *d* = 2.67
aWCT (%)	Male Female	70.3 ± 9.3 73.4 ± 3.4	68.4 ± 6.6 66.6 ± 3.9	61.6 ± 5.3 62.8 ± 6.1	*F*_(2,32)_ = 16.717, *p* < 0.001, ${\text{η}}_{\text{p}}^{2}$ = 0.511	*F*_(1,16)_ = 0.158, *p* = 0.697, ${\text{η}}_{\text{p}}^{2}$ = 0.010	1 vs. 2: *p* = 0.05, *d* = 1.08, 1 vs. 3: *p* < 0.001, *d* = 1.22, 2 vs. 3: *p* = 0.014, *d* = 2.62
5mRTT (s)	Male Female	5.57 ± 0.63 5.93 ± 0.23	5.38 ± 0.49 5.87 ± 0.33	5.69 ± 0.51 6.13 ± 0.36	*F*_(2,32)_ = 12.658, *p* < 0.001, ${\text{η}}_{\text{p}}^{2}$ = 0.442	*F*_(1,16)_ = 4.653, *p* = 0.047 ${\text{η}}_{\text{p}}^{2}$ = 0.225	1 vs. 2: *p* = 0.127, *d* = 0.46, 1 vs. 3: *p* < 0.001, *d* = 1.76, 2 vs. 3: *p* = 0.087, *d* = 0.60
5mOUT (s)	Male Female	2.65 ± 0.35 2.88 ± 0.2	2.58 ± 0.29 2.85 ± 0.21	2.73 ± 0.27 3.02 ± 0.21	*F*_(1.27, 20.32)_ = 31.336, *p* < 0.001, ${\text{η}}_{\text{p}}^{2}$ = 0.662	*F*_(1,16)_ = 4.842, *p* = 0.043, ${\text{η}}_{\text{p}}^{2}$ = 0.232	1 vs. 2: *p* = 0.016, *d* = 0.72, 1 vs. 3: *p* < 0.001, *d* = 2.40, 2 vs. 3: *p* = 0.004, *d* = 0.95
T_adapt_ (s)	Male Female	0.88 ± 0.06 0.89 ± 0.07	0.89 ± 0.07 0.92 ± 0.06	0.95 ± 0.04 0.96 ± 0.08	*F*_(2,32)_ = 15.135, *p* < 0.001, ${\text{η}}_{\text{p}}^{2}$ = 0.486	*F*_(1,16)_ = 0.435, *p* = 0.519, ${\text{η}}_{\text{p}}^{2}$ = 0.026	1 vs. 2: *p* = 0.387, *d* = 0.40, 1 vs. 3: *p* = 0.004, *d* = 0.91, 2 vs. 3: *p* < 0.001, *d* = 1.50
F_Peak_ (*N*)	Male Female	1,195 ± 416 1,245 ± 274	1,061 ± 359 1,044 ± 224	1,073 ± 347 952 ± 214	*F*_(2,32)_ = 27,309, *p* < 0.001, ${\text{η}}_{\text{p}}^{2}$ 0.631	*F*_(1,16)_ = 0.056, *p* = 0.816, ${\text{η}}_{\text{p}}^{2}$ = 0.003	1 vs. 2: *p* < 0.001, *d* = 1.27 , 1 vs. 3: *p* < 0.001, *d* = 0.46, 2 vs. 3: *p* < 0.001, *d* = 1.36
V_in_ (m/s)	Male Female	1.63 ± 0.16 1.5 ± 0.07	1.7 ± 0.1 1.54 ± 0.08	1.65 ± 0.13 1.48 ± 0.08	*F*_(1.21, 32)_ = 5.811, *p* = 0.021, ${\text{η}}_{\text{p}}^{2}$ = 0.266	*F*_(1,16)_ = 10.953, *p* = 0.004, ${\text{η}}_{\text{p}}^{2}$ = 0.406	1 vs. 2: *p* = 0.049, *d* = 0.64, 1 vs. 3: *p* < 0.001, *d* = 1.52, 2 vs. 3: *p* = 1, *d* = 0.04
V_exit_ (m/s)	Male Female	2.05 ± 0.34 1.76 ± 0.15	2.19 ± 0.29 1.83 ± 0.14	2.13 ± 0.31 1.77 ± 0.11	*F*_(2,32)_ = 14.321, *p* < 0.001, ${\text{η}}_{\text{p}}^{2}$ = 0.472	*F*_(1,16)_ = 9.934, *p* = 0.006, ${\text{η}}_{\text{p}}^{2}$ = 0.383	1 vs. 2: *p* < 0.001, *d* = 1.27 , 1 vs. 3: *p* = 0.149, *d* = 0.73, 2 vs. 3: *p* = 0.027, *d* = 0.44
Manipulating Tuck Index		Close	Reference	Far			
Tuck index	Male Female	0.44 ± 0.1 0.44 ± 0.09	0.61 ± 0.06 0.68 ± 0.05	0.78 ± 0.06 0.85 ± 0.07	*F*_(2, 22)_ = 88.594, *p* < 0.001, ${\text{η}}_{\text{p}}^{2}$ = 0.89	*F*_(1,16)_ = 3.122, *p* = 0.105, ${\text{η}}_{\text{p}}^{2}$ = 0.221	1 vs. 2: *p* < 0.001, d =1.39, 1 vs. 3: *p* < 0.001, d =2.36, 2 vs. 3: *p* < 0.001, *d* = 3.55
5mRTT (s)	Male Female	5.9 ± 0.64 6.5 ± 0.31	5.39 ± 0.49 5.89 ± 0.34	5.66 ± 0.55 6.28 ± 0.25	*F*_(2,32)_ = 77.757, *p* < 0.001, ${\text{η}}_{\text{p}}^{2}$ = 0.829	*F*_(1,16)_ = 8.309, *p* = 0.011, ${\text{η}}_{\text{p}}^{2}$ = 0.342	1 vs. 2: *p* < 0.001, *d* = 2.07, 1 vs. 3: *p* = 0.001, *d* = 13.65, 2 vs. 3: *p* < 0.001, *d* = 12.99
WCT (s)	Male Female	0.54 ± 0.14 0.61 ± 0.21	0.36 ± 0.04 0.31 ± 0.05	0.25 ± 0.05 0.2 ± 0.04	*F*_(1.13, 18.02)_ = 57.0, *p* < 0.001, ${\text{η}}_{\text{p}}^{2}$ = 0.781	*F*_(1,16)_ = 0.125, *p* = 0.728, ${\text{η}}_{\text{p}}^{2}$ = 0.008	1 vs. 2: *p* < 0.001, *d* = 1.38, 1 vs. 3: *p* < 0.001, *d* = 2.05, 2 vs. 3: *p* < 0.001, *d* = 1.96
Initiation distance (m)	Male Female	0.87 ± 0.07 0.8 ± 0.09	1.06 ± 0.09 1.07 ± 0.07	1.32 ± 0.16 1.35 ± 0.07	*F*_(1.41, 22.73)_ = 360.9, *p* < 0.001, ${\text{η}}_{\text{p}}^{2}$ = 0.956	*F*_(1,16)_ = 0.076, *p* = 0.786, ${\text{η}}_{\text{p}}^{2}$ = 0.005	1 vs. 2: *p* < 0.001, *d* = 3.45, 1 vs. 3: *p* < 0.001, *d* = 3.75, 2 vs. 3: *p* < 0.001, *d* = 4.59
F_Peak_ (*N*)	Male Female	934 ± 328 841 ± 195	1047 ± 352 1044 ± 224	1293 ± 521 1251 ± 257	*F*_(1.37, 21.913)_ = 56.926, *p* < 0.001, ${\text{η}}_{\text{p}}^{2}$ = 0.781	*F*_(1,16)_ = 13.346, *p* = 0.74, ${\text{η}}_{\text{p}}^{2}$ = 0.007	1 vs. 2: *p* < 0.001, *d* = 1.61, 1 vs. 3: *p* < 0.001, *d* = 1.26, 2 vs. 3: *p* < 0.001, *d* = 2.34
V_in_ (m/s)	Male Female	1.63 ± 0.11 1.48 ± 0.07	1.69 ± 0.11 1.54 ± 0.09	1.62 ± 0.12 1.46 ± 0.09	*F*_(1.381, 22.095)_ = 10.522, *p* = 0.002, ${\text{η}}_{\text{p}}^{2}$ = 0.397	*F*_(1,16)_ = 13.346, *p* = 0.002, ${\text{η}}_{\text{p}}^{2}$ = 0.455	1 vs. 2: *p* = 0.001, *d* = 1.09, 1 vs. 3: *p* = 0.013, *d* = 0.82, 2 vs. 3: *p* = 1.0, *d* = 0.22
5mOUT (s)	Male Female	2.97 ± 0.32 3.09 ± 0.35	2.68 ± 0.28 2.8 ± 0.3	2.86 ± 034 2.94 ± 0.27	*F*_(2,32)_ = 25.935, *p* < 0.001, ${\text{η}}_{\text{p}}^{2}$ = 0.618	*F*_(1,16)_ = 0.599, *p* = 0.45, ${\text{η}}_{\text{p}}^{2}$ = 0.036	1 vs. 2: *p* < 0.001, *d* = 1.56, 1 vs. 3: *p* = 0.003, *d* = 0.95, 2 vs. 3: *p* = 0.006, *d* = 0.91

*The last column represents the results of the post-hoc testing (p-values and Cohen's d). ${\text{η}}_{\text{p}}^{2}$ = partial eta squared, 1 = Reference, 2 = Short / Close, 3 = Long/ Far. The data of the reference trials from the Manipulating WCT study vary slightly from those of the Manipulating Tuck Index study due to the different number of included trials*.

There was a significant main effect of the WCT manipulation on the 5mRTT. *Post-hoc* testing revealed that the swimmers achieved shorter 5mRTT in the reference condition when compared to the long WCT condition, in the absence of any other significant differences. Overall, the 5mRTT was significantly shorter for the male swimmers than the female swimmers. However, there was no significant sex by condition interaction, implying that the differences in the 5mRTT between the conditions were not biased by the differences between sexes.

By splitting up the turn action into approach and exit components, the results revealed that T_adapt_ differed significantly between the conditions, with a longer T_adapt_ for the long WCT trials when compared to the reference and short contact trials. Furthermore, there was a significant effect of condition on V_in_. During the short and long WCT conditions the V_in_ was significantly lower compared to V_in_ in the reference condition, while in all conditions the speed was significantly higher for the male swimmers than for the female swimmers. A similar effect of WCT was found in the 5mOUT, with the reference trials showing the lowest 5mOut time and all experimental conditions being significantly different from each other.

The best fitting linear mixed effect model (AIC: −150.99, LL: 110.5, Intercept: 3.12, *p* < 0.001) involved significant negative effects of WCT (*p* < 0.001), F_Peak_ (*p* = 0.04), V_in_ (*p* = 0.02), T_adapt_ (*p* = 0.002), and V_exit_ (*p* < 0.001) on the 5mRTT, resulting in the following model equation:

5mRTT = 3.1231–4.2276 × WCT−2.1799 × F_Peak_-4.8258 × V_in_-2.677 × T_adapt_-9.5239 × V_exit_ +ε

The ICC and Bland-Altman (BA) values were as follows [bias (95 % CI), LoAL = Lower level of agreement (95 % CI), LoAU: Upper level of agreement (95 % CI)]:

Reference vs. Short: ICC: *r* = 0.96, *p* < 0.001, BA: bias: −0.061 [−0.08; −0.04], LoAL: −0.128 [−0.16; −0.10], LoAU: 0.006 [−0.02; 0.04].

Reference vs. Long: ICC: *r* = 0.85, *p* < 0.001, BA: bias: −0.102 [−0.13; −0.08], LoAL: −0.198 [−0.24; −0.16], LoAU: −0.006 [−0.05; 0.04].

Long vs. Short: ICC: *r* = 0.78, *p* = 0.002, BA: bias: −0.163 [−0.19; −0.13], LoAL: −0.28 [−0.34; −0.23], LoAU: −0.043 [−0.10; 0.01].

These values correspond to good to excellent reliability concerning the consistency and a good agreement between conditions (Haghayegh et al., 2020).

### Manipulation of tuck index

In total, 287 out of 306 tumble turn trials were included to examine the effect of the Tuck Index on turn performance. The detailed statistical results are reported in Table 3. The realized Tuck Indices were significantly different across the experimental conditions in the absence of a significant effect of sex. The *post-hoc* tests revealed that the Tuck Index in the reference condition was significantly lower compared to the far condition and significantly higher compared to the close condition, indicating that also this manipulation was successful.

There was a significant effect of condition on the 5mRTT. *Post-hoc* tests revealed that performance was significantly better in the reference condition than in the far and close condition, respectively. Male swimmers were significantly faster than female swimmers in the absence of a significant interaction involving sex. The experimental manipulation of the Tuck Index also affected the WCT. The *post-hoc* tests revealed that the WCT significantly decreased from the close to the reference to the far condition. During the far condition, the swimmers generated the highest F_Peak_ while it was lowest in the close condition.

The experimental manipulation affected both the approach and exit components of the turn. A significant effect was found for the turn initiation distance, because the initiation of the turn in the reference condition occurred significantly further from the wall compared to the close condition and significantly closer to the wall compared to the far condition. Moreover, turning during the far condition occurred significantly further from the wall compared to the close condition. Also, a significant main effect of the condition was found for V_in_. *Post-hoc* testing revealed that V_in_ was significantly higher in the reference condition than in the close and far condition. There was no significant difference between the far and close condition. Male swimmers were significantly faster before the turn compared to female swimmers. This indicates that the differences in turn performance in the reference, far and close trials might have been attributable to a change in speed before the turn. The 5mOUT was used to examine whether the turn was different between the trials, independent of the swimming velocity before the turn. A significant main effect of the condition was found for the 5mOUT. *Post-hoc* testing showed that the 5mOUT was significantly shorter for the reference condition compared to both the far and close conditions, while the far condition resulted in significantly a shorter 5mOUT time when compared to the close condition. This indicates that the turn was indeed different between the experimental conditions, independent of the swimming speed before the turn.

A negative correlation between Tuck Index and WCT (*r* = −0.830, *p* < 0.001) and a positive correlation between Tuck Index and F_Peak_ were found (*r* = 0.473, *p* < 0.001), in the absence of a significant correlation between Tuck Index and 5mRTT (*r* = −0.102, *p* = 0.100).

**Figure 4:**
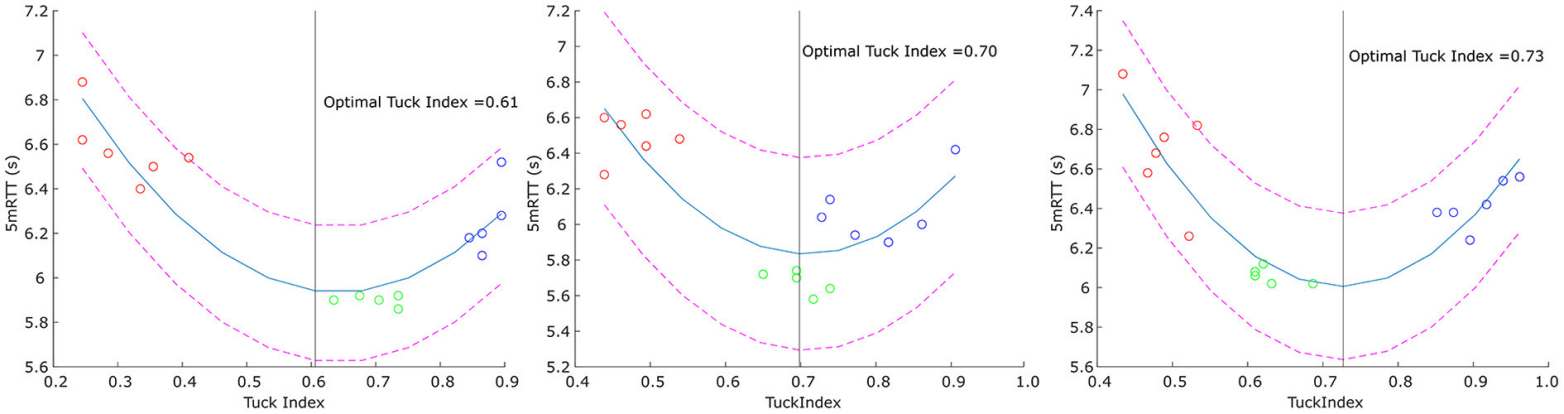
Prediction of the optimal Tuck Index for three selected swimmers. Red circles: Close condition trials, green circles: Reference condition trials, blue circles: Far condition trials. Pink dashed lines: 95 % prediction interval, black line: optimal Tuck Index.

For each participant, a prediction model was made for the optimal Tuck Index using a quadratic estimation function based on the 5mRTT (see Figure 4) and 5mOUT. These optimal Tuck Indices were compared with the participant's best trial to arrive at an individual advice as to how their turn performance might be improved. The intra-individual mean Tuck Index during the reference trials was 0.65 ± 0.06, while the predicted Tuck Index based on the 5mRTT and 5mOUT was estimated to be 0.70 ± 0.04 (range 0.64 and 0.77) and 0.69 ± 0.07 (range 0.58 and 0.83), respectively.

The ICC and Bland-Altman (BA) values were as follows [bias (95 % CI), LoAL = Lower level of agreement (95 % CI), LoAU: Upper level of agreement (95 % CI)]:

Reference vs. Close: ICC: *r* = 0.41, *p* = 0.14, BA: bias: −0.1661 [−0.23; −0.11], LoAL: −0.40 [−0.50; −0.30], LoAU: 0.068 [−0.04; 0.17].

Reference vs. Far: ICC: *r* = 0.68, *p* = 0.01, BA: bias: 0.207 [0.16; 0.25], LoAL: 0.035 [−0.04; 0.11], LoAU: 0.379 [0.30; 0.46].

Far vs. Close: ICC: *r* = 0.36, *p* = 0.19, BA: bias: 0.373 [0.32; 0.43], LoAL: 0.167 [0.08; 0.26], LoAU: 0.579 [0.49; 0.67].

These values correspond to a low to moderate reliability with regard to consistency and a moderate agreement between conditions (Haghayegh et al., 2020).

## Discussion

In the present study, the effects of experimentally induced changes in the WCT and Tuck Index on the 5mRTT and other performance-related variables were examined. The results showed that the swimmers were able to change the two performance-related variables and that these changes affected their turn performance, as well as other performance-related variables, notably F_max_ and V_exit_. Although on average the tumble turn performance was best in the reference trials compared to the manipulated trials, detailed statistical analyses of the experimentally induced variations in the performance-related variables of interest revealed that prolonging the WCT and adopting a Tuck Index of about 0.7 might help to improve the tumble turn performance.

### Manipulation of WCT

The WCT and F_Peak_ results of the reference trials in this study were comparable to those of previous tumble turn studies (Lyttle et al., 1999; Puel et al., 2012; Cossor et al., 2014; Skyriene et al., 2017). The 5mRTT was shortest during the reference trials and longer for both the short and long WCT trials. This seems to contradict the results of the derived linear mixed model in which, as hypothesized, the WCT and the 5mRTT were negatively correlated. This apparent contradiction may be explained by other mediating variables of the turning performance. Nicol et al. included several performance-determining variables in their analysis and were unable to identify a single strong performance predictor. Based on this result, they concluded that it is necessary to adopt a holistic approach in which the turn technique is changed to examine the impact of these changes on performance (Nicol et al., 2019). By adopting such an approach in the present study we found that WCT, F_Peak_, T_adapt_, V_in_, and V_exit_ all contribute significantly to the 5mRTT. Manipulating the WCT affected the F_Peak_ and V_exit_ with a shorter WCT resulting in a higher F_Peak_, and a longer WCT accompanying a higher V_exit_, which is consistent with previous findings (Lyttle et al., 1999; Klauck, 2005), as well as our hypotheses. The high F_Peak_ during a short WCT could have been caused by a high impact force, resulting in a less efficient push-off and finally a lower V_exit_ (Lyttle et al., 1998). The increased V_exit_ during the longer WCT is likely related to the later occurrence of F_Peak_ (Lyttle et al., 1999; Puel et al., 2012), which increases the acceleration during push-off due to the more streamlined position at the end of the WCT (Klauck, 2005; Puel et al., 2012). However, to have a beneficial effect on the tumble turn performance, the higher V_exit_ has to compensate for the time lost due to the longer WCT. Other relevant mediating variables are the glide depth, the initiation time of the dolphin kicks (Cossor et al., 2014) and the point of resurfacing (30), which were not taken into account in the present study. However, their impact on the tumble turn performance might nevertheless be limited according to Blanksby et al. (1996) and Nicol et al. (2019).

The swimmers in the present study further showed a decrease in the active WCT (aWCT) with increasing WCT, which might indicate that they had difficulty adapting to the task and could have made more efficient use of the time on the wall. The challenge of maintaining the same performance level while changing the WCT is also reflected in the adaptation time, which increased during the long WCT trials. To conclude, the results indicate that the WCT has to be sufficiently long to bring the body into a properly streamlined position in order to make optimal use of a powerful push-off force.

### Manipulation of tuck index

Also, the Tuck Indices determined in this study are in line with those reported in previous studies, i.e., 0.61 ± 0.1 and 0.68 ± 0.05 for female and male swimmers in the present study vs. 0.56 ± 0.1 and 0.71 ± 0.09 in previous studies (Blanksby et al., 1996; Skyriene et al., 2017). On average, the 5mRTT was the fastest in the reference trials, compared to both the close and far conditions. The Tuck Index was negatively correlated with the WCT because it takes time to extend the legs. This finding is also in line with the results of previous studies (Cossor et al., 1999; Blanksby et al., 2004). In addition, the Tuck Index correlated positively with F_Peak_, which is consistent with the results of Blanksby et al. (2004). This can be explained by the small knee flexion angle during close turns, which forces the extensor muscles to work at an inefficient length, thus producing less force compared to a larger knee flexion angle. Pereira et al. advised a knee angle between 110 and 120° for optimal turn performances (Pereira et al., 2006). Although it is strictly speaking inaccurate to directly translate knee angles into a Tuck Index, one may assume that the distance between the trochanter major and the wall is mainly influenced by the knee flexion angle, given that the sagittal plane is the main plane of movement. Based on this assumption, the estimated optimal Tuck Index of 0.70 would result in a knee flexion angle of 90°. This value is well below the range of knee joint angles suggested by Pereira et al. (2006). However, the methods used in the present study were quite different from the one used by Pereira et al. The optimal Tuck Index of 0.7, and hence the corresponding knee flexion angle of 90°, is the result of a quadratic model estimation and reflects the optimal value across all participants in this study. Pereira et al., in contrast, included only the preferred knee flexion angles that ranged between 29 and 161° and correlated them with the corresponding turn times. Hence, it might well be, that their participants did not make use of the optimal knee flexion angle to achieve the best turn performance. Also, they used a 7.5mRTT instead of the 5mRTT used in this study (Pereira et al., 2006). Interestingly, the knee joint angle of 90° that we arrived at is in line with the optimal angle for on-land squat jumps (Mitchell et al., 2017; Janicijevic et al., 2020). Even though the push-off from the wall is similar to vertical on-land squat jumps, little is known about the impact of body orientation (horizontal vs. vertical) and the drag force of the water (vs. the gravitational force on land) (Nicol et al., 2019). However, the push-off force and the force profile during wall push-off were found to be comparable to on-land squat jumps during maximum wall push-off and vertical on-land squat jumps (Guignard et al., 2017).

The optimal Tuck Index, as a result of the quadratic optimization function, was 0.70 ± 0.04. Prins and Patz also estimated the optimal Tuck Index and reported a value of 0.46 for achieving a maximum push-off velocity. However, as they acknowledged themselves, this might not result in an optimal round-trip time (Prins and Patz, 2006). Ultimately, the swimmer wants to swim as fast as possible; we therefore based the calculations on the 5mRTT. For some swimmers, this meant that their optimal Tuck Index fell within their reference values, while the prediction of the Tuck Index was higher than the reference values for some of the other swimmers (Figure 4), indicating that they might be able to turn faster when turning slightly further from the wall. This result illustrates the practical value of prediction models in seeking to optimize the performance of individual swimmers. Elite athletes are striving to optimize their performance in every possible way. Improving the turning technique holds great potential for improving swimming success due to the high contribution of the turn to the overall swim time. Also, elite swimmers have highly individual requirements and adjusting one of the determinants might result in an advantage for one swimmer and a disadvantage for another. By investigating the data on an individual level, such individual differences are taken into account. However, the predicted optimal Tuck Indices might be biased by the distance between the experimental conditions. Swimmers showing unequal differences between the three Tuck Index conditions will result in a shifted quadratic function compared to swimmers with balanced differences (Figure 4). As most swimmers showed greater differences between the close and the reference condition compared to the differences between the reference to the far condition, the optimal Tuck Index could therefore underestimate the true optimum.

In the current study, no significant relationship was found between the Tuck Index and 5mRTT, while in previous studies both positive (Cossor et al., 2014; Skyriene et al., 2017) and negative relationships (Blanksby et al., 2004) were reported. Our findings provide an explanation for these apparently contradictory results. Notably, the studies that found a positive relationship reported Tuck Indices slightly higher (Cossor et al., 2014; Skyriene et al., 2017) than the optimal Tuck Index of 0.7 derived in this study, whereas the studies that found a negative relationship reported Tuck Indices that were lower than 0.7. Including Tuck Indices that are both below and above the optimal value of 0.7 allowed us to identify the real relationship between the Tuck Index and the tumble turn performance, which is not linear, but quadratic.

The intra-class correlation between the different conditions revealed that the consistency of the Tuck Indices was only moderate. This could indicate that the swimmers had difficulty to consistently execute repeatable Tuck Indices within the manipulated conditions. This was not the case for the manipulation of the WCT. This result could have been anticipated considering that the adaptation of the Tuck Index represents a major invasion into the swimmer's preferred turn technique. However, this also implies that practicing the non-reference conditions for the Tuck Index could further increase the accuracy of the prediction model.

### Limitations

The Tuck Index data of five swimmers were missing due to missing video recordings. The repeated measures ANOVA of the tuck index and the polyfit results were thus only performed with the data of 13 participants, that is, with a reduced sample size.

During the manipulation of the WCT and the Tuck Index, the swimmers were instructed to keep the approach of the wall and underwater phase as constant as possible. However, this was not the case for V_in_ and T_adapt_. The slower V_in_ in the manipulated conditions might have been caused by the fact that the participating swimmers were less familiar with the experimental conditions than with the reference conditions. Additionally, the onset of fatigue might have played a role as well, even though the participants were allowed as much time as they liked to recover between trials. The swimmers indicated that they felt tired toward the end of the session, which might have affected the performance of the trials at the end of the day. This was reflected in a lower 5mRTT and V_in_ for the trials in question. Moreover, prior to the experiment, the swimming pool was closed due to COVID-19 regulations. It might have been the case that this interruption of the regular training schedule affected the overall performance when compared to regular training times. However, all included athletes were Dutch national-level swimmers, which might have mitigated the effect of the training situation on the reported outcomes.

The criteria for a valid trial were chosen in such a way that the manipulations resulted in distinct WCTs or Tuck Indices for each of the conditions. However, this resulted in a lack of information about the effect of those WCTs or Tuck Indices that range between the defined conditions, while it might be that the swimmer would swim their fastest turn performance in that range. Although it would have been beneficial to also cover these values, the number of trials was already high and a further increase would have most certainly led to fatigue.

## Conclusion

To increase their tumble turn performance swimmers are recommended to focus on generating a high F_Peak_ at the end of the WCT when the body is in a properly streamlined position. To this end, a sufficiently long WCT is required. The present analyses further suggest that it is possible to recommend an optimal Tuck Index for individual swimmers, which might help to improve their tumble turn and thus their race performance. Further, the presented approach to estimate the optimal individual Tuck Index is readily applicable to a training session. Coaches and swimmers could therefore test whether an adaptation of the Tuck Index is indicated to improve the turn performance. Also, these two variables are readily adaptable by swimmers, whereas this might be more difficult for other variables. The present results need to be confirmed by an intervention study in which swimmers are trained to perform the tumble turn with the recommended WCTs and Tuck Indices.

## Data availability statement

The datasets presented in this article are not readily available because they contain the data of elite athletes which might be identifyable if publishing the information. Requests to access the datasets should be directed to s.david@vu.nl.

## Ethics statement

The studies involving human participants were reviewed and approved by the Scientific and Ethical Review Board (VCWE) of the Faculty of Behavioural and Movement Sciences of the Vrije Universiteit Amststerdam. Written informed consent to participate in this study was provided by the participants' legal guardian/next of kin.

## Author contributions

PB, TG, and MD conceptualized and designed the study. SD, TG, and MD performed the literature search. TG, MD, and PK were involved in data collection and performed the data processing. SD and PB drafted the current manuscript. All authors substantially contributed to the interpretation of the data, revised it critically, and approved the final version of the manuscript.

## Conflict of interest

The authors declare that the research was conducted in the absence of any commercial or financial relationships that could be construed as a potential conflict of interest.

## Publisher's note

All claims expressed in this article are solely those of the authors and do not necessarily represent those of their affiliated organizations, or those of the publisher, the editors and the reviewers. Any product that may be evaluated in this article, or claim that may be made by its manufacturer, is not guaranteed or endorsed by the publisher.
